# Impact of COVID-19 Public Health Measures on Antiretroviral Therapy Use Among Ugandans Living with HIV in Sero-Different Couples

**DOI:** 10.1007/s10461-025-04612-2

**Published:** 2025-01-10

**Authors:** Timothy R. Muwonge, Erika Feutz, Rogers Nsubuga, Jane M. Simoni, Florence Nambi, Lylianne Nakabugo, Sylvia Namanda, Joseph Kibuuka, Dorothy Thomas, Ingrid T. Katz, Katherine K. Thomas, Norma C. Ware, Monique A. Wyatt, Herbert Kadama, Andrew Mujugira, Renee Heffron

**Affiliations:** 1 Infectious Diseases Institute, Makerere University, Kampala, Uganda; 2 Department of Global Health, University of Washington, Seattle, WA USA; 3 Department of Psychology, University of Washington, Seattle, WA USA; 4 Brigham and Women’s Hospital, Boston, MA USA; 5 Division of Global Health Equity, Department of Medicine, Brigham and Women’s Hospital, Boston, MA USA; 6 Department of Global Health and Social Medicine, Harvard Medical School, Boston, MA USA; 7 Ministry of Health, Box 7272, Kampala, Uganda; 8 Department of Medicine, University of Alabama at Birmingham, 845 19th Street South, Bevill Biomedical Research Building, Room 256D, Birmingham, AL 35294-2170 USA; 9 CharacterStrong LLC Seattle, Washington, USA

**Keywords:** HIV, ART Initiation, Viral load suppression, COVID-19

## Abstract

Antiretroviral therapy (ART) use and HIV suppression among people living with HIV (PLHIV) are critical for HIV control and prevention. Extreme restrictions on movement early during the COVID-19 pandemic in Uganda may have impeded the ability to initiate and sustain access to and use of ART. From our stepped-wedge cluster-randomized trial of an integrated PrEP and ART intervention for HIV-serodifferent couples at 12 ART clinics in Uganda, we identified participants who enrolled and had a 6-month post-ART initiation viral load measured before the beginning of the first COVID-19 lockdown (Period 1), participants whose enrollment and 6-month viral load measurement straddled pre-COVID and COVID lockdown times (Period 2), and participants whose enrollment and 6-month viral load were quantified entirely during COVID-19 (Period 3). ART and viral load data were abstracted from standard-of-care HIV clinic records. We used adjusted generalized estimating equation models to compare viral suppression between the different periods. We enrolled 1,381 PLHIV, including 896 (64.9%) in Period 1, 260 (18.8%) in Period 2, and 225 (16.3%) in Period 3. Almost all (1371, 99.3%) initiated ART within 90 days of enrollment and 59.2% had baseline CD4 > 350 cells/mm^3^. Among those enrolled, 88.8% of participants in Period 1 were virally suppressed (< 1000 copies/mL) within six months of ART initiation, 80.5% in Period 2, and 88.2% in Period 3. All pairwise comparisons demonstrated statistically similar levels of viral suppression. Despite COVID-19 lockdown measures, PLHIV in serodifferent partnerships successfully initiated ART and attained and maintained viral suppression.

## Introduction

The Coronavirus Disease (COVID-19) pandemic and associated mitigation measures impacted healthcare access globally, including services for chronic conditions, such as HIV [1]. In Uganda, strict curfews and restrictions on public or private transport [2] limited people’s ability to travel to facilities and access HIV care services [3, 4]. Before the COVID-19 pandemic, access to HIV treatment in Uganda required physical attendance at a health facility, and there were concerns that COVID-19 public health regulations would impact access to HIV treatment and rates of viral suppression, in the immediate as well as the long-term [5, 6]. If extensive enough, these gaps could ultimately result in associated morbidity, and onward HIV transmission, two downstream effects of unsuppressed HIV viremia [7].

When the pandemic began, we were implementing the Partners PrEP Program (PPP), a stepped-wedge cluster randomized trial (clinicaltrials.gov # NCT03586128) aimed at evaluating the integration of HIV pre-exposure prophylaxis (PrEP), into HIV treatment services for HIV serodifferent couples in 12 health facilities in Kampala and Wakiso Districts, Uganda [8]. Leveraging data from this program, we evaluated data describing antiretroviral therapy (ART) initiation and HIV viral load among people living with HIV (PLHIV) who were in known serodifferent relationships in the periods before, during, and following COVID-19 lockdown measures in Uganda.

## Methods

### Study Design and Procedures

The Partners PrEP Program was implemented between 2017 and 2021 in 12 public health clinics in Kampala, Uganda, encompassing 3 steps with 4 clinics initiating PrEP delivery at each step. For this analysis, we utilized secondary data from March 2020 to December 2021 from the sample of enrolled HIV serodifferent couples who had been recruited from HIV testing sites in Kampala and Wakiso districts. For partners living with HIV, antiretroviral treatment (ART) was offered immediately upon diagnosis and at every encounter thereafter. HIV viral load was quantified approximately 6 months after ART initiation as standard of care at the Uganda Central Public Health Laboratory (Abbott m2000 Real-Time HIV-1, Abbott Park, Illinois, USA or Roche COBAS® Ampli Prep v 2.0 assay, Roche Diagnostics, Indianapolis, Indiana, USA) [9]. The study launched delivery of PrEP for partners without HIV according to the national guidelines [10], facilitating the availability of PrEP within the ART clinic and specifically for members of HIV serodifferent couples. Thus, the timing of PrEP availability was determined by the study randomization schema, since PrEP was not widely available to HIV-negative partners in serodifferent relationships at the time. Data on demographics, HIV status, ART dispensing, and HIV viral load, were abstracted from medical records at the health facilities into a web-based data collection system using tablets [11].

### Outcomes

For this analysis, ART initiation was defined as the earliest date of ART dispensation in the medical records. Viral suppression was defined as having HIV RNA < 1000 copies/mL [10] assessed between 42 and 270 days from ART initiation. Participants who did not initiate ART or who initiated ART > 90 days prior to enrollment were categorized into COVID-19 impact categories using the date six months from enrollment and assumed to be unsuppressed. Participants without a viral load assessment between 42 and 270 days from ART initiation were excluded.

### Data Analysis

We assessed rates of viral suppression for 3 different periods related to the COVID-19 pandemic using 18 March 2020 as the first date when Uganda's public health restrictions began [12]. Period 1 included participants who were enrolled and had a 6-month viral load assessment before 18 March (entirely before COVID-19). Period 2 included participants who enrolled before 18 March and whose 6-month viral load assessment was measured after 18 March (straddling pre-COVID and early COVID periods). Period 3 included participants who enrolled after 18 March and had a 6-month viral load assessment quantified thereafter (entirely during COVID-19). We applied a generalized estimating equation model with binomial outcome and log link to generate all pairwise comparisons of viral suppression across the different periods. The relative risks describing the association between COVID-19 time periods and viral suppression were calculated using separate generalized estimating equations models. All models were adjusted for confounding by PrEP implementation status and clustering by clinic and correcting for small cluster sizes. Analysis was conducted using R version 3.6.0 [13].

### Ethical Considerations

The protocol was reviewed and approved by the Uganda National HIV/AIDS Research Committee (ARC 194), the Uganda National Council for Science and Technology (HS 2381), and the University of Washington Human Subjects Division (STUDY00000320). Local administration approval to access de-identified data was obtained from Kampala Capital City Authority and Wakiso District. Thus, individual consent was not required. An independent data monitoring committee reviewed study execution and feasibility annually.

## Results

We enrolled 1,381 partners living with HIV, including 896 (64.9%) in Period 1, 260 (18.8%) in Period 2, and 225 (16.3%) in Period 3 (Table 1). Median age was 28 years (interquartile range [IQR] 23.2, 34), 88.6% were married or living with a partner, and 849 (61.5%) were female, of whom nearly half (49%) were pregnant. Almost all participants with HIV (99.3%) initiated ART within 90 days of enrollment, and more than half (59.2%) had CD4 > 350 cells/mm^3^ at enrollment.

**Table 1:** Baseline characteristics by COVID-19 impact category for partners living with HIV

	Period 1	Period 2	Period 3	Total
Participants enrolled	896	260	225	1381
Age in years (median (IQR))	28 (23.9, 34)	26 (22.3, 31.2)	28 (24, 35)	28 (23.2, 34)
Female sex	537 (59.9%)	172 (66.2%)	140 (62.2%)	849 (61.5%)
Pregnant	202/397 (50.9%)	75/167 (44.9%)	63/130 (48.5%)	340/694 (49%)
Marital status (N)	771	224	190	1185
Never Married	63 (8.2%)	16 (7.1%)	10 (5.3%)	89 (7.5%)
Married	596 (77.3%)	172 (76.8%)	151 (79.5%)	919 (77.6%)
Partnered, living together	78 (10.1%)	33 (14.7%)	20 (10.5%)	131 (11.1%)
Divorced	32 (4.2%)	3 (1.3%)	9 (4.7%)	44 (3.7%)
Widowed	2 (0.3%)	0 (0%)	0 (0%)	2 (0.2%)
Contraceptive use (N)	787	258	219	1264
Condoms	42 (5.3%)	3 (1.2%)	13 (5.9%)	58 (4.6%)
Oral contraceptive pills	5 (0.6%)	1 (0.4%)	0 (0%)	6 (0.5%)
IUD/injectable/implantable	32 (4.1%)	10 (3.9%)	6 (2.7%)	48 (3.8%)
Diaphragm/cervical cap	0 (0%)	0 (0%)	0 (0%)	0 (0%)
Vasectomy/tubal ligation	0 (0%)	0 (0%)	0 (0%)	0 (0%)
None	708 (90%)	244 (94.6%)	200 (91.3%)	1152 (91.1%)
CD4 + count, cells/mm^3^ (N)	541	120	52	713
< 200	104 (19.2%)	20 (16.7%)	8 (15.4%)	132 (18.5%)
200–349	121 (22.4%)	26 (21.7%)	10 (19.2%)	157 (22%)
350–499	116 (21.4%)	23 (19.2%)	10 (19.2%)	149 (20.9%)
500+	198 (36.6%)	51 (42.5%)	24 (46.2%)	273 (38.3%)
WHO HIV stage (N)	873	256	222	1351
Stage 1–2	810 (92.8%)	243 (94.9%)	213 (95.9%)	1266 (93.7%)
Stage 3–4	63 (7.2%)	13 (5.1%)	9 (4.1%)	85 (6.3%)
Days from HIV diagnosis to enrollment [median (IQR)]	0 (0, 0)	0 (0, 1)	0 (0, 3)	0 (0, 0)
ART initiated	887 (99%)	259 (99.6%)	225 (100%)	1371 (99.3%)

Across periods, 88.8% were virally suppressed within 6 months of ART initiation in period 1, 80.5% in period 2, and 88.2% in period 3 (Table 2). Pairwise comparisons showed no statistically significant differences in viral suppression across periods (for period 2 vs. 1: relative risk [RR} = 0.97, *p* = 0.61, test statistic = − 0.52; for period 3 vs. 1, RR = 1.08, *p* = 0.11, test statistic = 1.86; and for period 3 vs. 2: RR = 1.11, *p* = 0.18, test statistic = 1.45) (Table 2). The median time from ART initiation to viral load assessment in periods 1, 2 and 3 were 128 days (IQR 95–173), 175.5 days (IQR 146–206.5), and 130.5 days (IQR 97.8–168.3).

**Table 2:** Summary of 6-month viral suppression, missing VL results, and reason reported for missing COVID-19 impact category

	Period 1	Period 2	Period 3	Total
Total of VL suppression	604 (88.8%)	95 (80.5%)	127 (88.2%)	826 (87.7%)
Time (days) from ART initiation to VL measurement (median [IQR])	128 (95, 173)	175.5 (146, 206.5)	175.5 (146, 206.5)	136 (97, 177)
Proportion missing to VL measurement^a^	271 (30%)	88 (34%)	80 (36%)	439 (32%)
Reason for missingness to VL measurement				
Transfer	112 (41.3%)	33 (37.5%)	9 (11.2%)	154 (35.1%)
No follow-up visit	72 (26.6%)	17 (19.3%)	9 (11.2%)	98 (22.3%)
Client died	10 (3.7%)	4 (4.5%)	0	14 (3.2%)
No blood draw	10 (3.7%)	7 (8%)	0	17 (3.9%)
Results not available	5 (1.8%)	2 (2.3%)	1 (1.2%)	8 (1.8%)
VL conducted > 270 days after initiation	25 (9.2%)	16 (18.2%)	4 (5%)	45 (10.3%)
No reason	37 (13.7%)	9 (10.2%)	57 (71.2%)	103 (23.5%)
Statistical comparisons of effect on viral suppression**	Period 2 vs. Period 1	Period 3 vs. Period 1	Period 3 vs. Period 2	
Relative risk (95% confidence interval)	0.97 (0.86–1.10)	1.08 (0.98–1.19)	1.11 (0.94–1.31)	
Test statistic (*p*-value)	− 0.52 (0.613)	1.86 (0.107)	1.45 (0.183)	

**GEE model adjusted for PrEP implementation

^a^Percentage of participants missing a viral load result out of those enrolled

From ART initiation to VL assessment dates, 439 people (32%) missed their VL assessment. The most common reasons for missing VL data were facility transfer (35%), lack of follow-up visits (22.3%), VL conducted later than 270 days after initiation (usually from 9 to 12 months after initiation) (10.3%), and no blood draw conducted (3.9%).

## Discussion

The COVID-19 pandemic catalyzed unprecedented disruptions across global health systems, profoundly affecting access to essential healthcare services, including those for chronic conditions like HIV. However, our data indicate that despite COVID-19 lockdown measures, PLHIV initiated ART and achieved similar viral suppression levels before and after strict public health measures were taken, as noted by the lack of difference in rates of viral suppression across the 3 periods. The small non-statistically significant decline in viral suppression experienced in the immediate wake of COVID-19 restrictions (period 2) was not maintained. In the case of pandemics or other calamities that involve restrictions to public movement and transport, it is possible to maintain pre-pandemic rates of viral suppression even with lockdown measures.

In Uganda, where approximately 30% of new HIV infections occur among heterosexual serodifferent couples [14–17], effective ART remains a cornerstone intervention in reducing transmission risk. Our findings indicate a resilient response to the challenges posed by COVID-19 restrictions. Despite limitations in public transportation and strict curfews, the vast majority (99.3%) of individuals living with HIV initiated ART within 90 days of enrolment, underscoring the commitment of both PLHIV and healthcare providers to ensure continued access to treatment [6, 18].

Several factors may have contributed to the observed trends in ART initiation and viral suppression during the COVID-19 pandemic. Healthcare facilities quickly adapted service delivery mechanisms, including adding telemedicine consultations, extended clinic hours, and decentralized medication distribution points referral to nearby facilities for refill, which helped alleviate barriers to care access imposed by transportation restrictions and curfews [19, 20].

A previous study depicted that the COVID-19 lockdown negatively impacted ART initiation [5], contrary to the findings of our study, which showed no difference. In 2021, a study in Uganda showed similar results: no statistically significant change in electronically measured adherence or viral suppression [20]. Related studies reported moderate to high viral suppression rates pre-lockdown that remained steady throughout the post-lockdown period [5, 21, 22]. These findings compare well with ours, which showed no significant change in viral suppression compared to pre-pandemic, pandemic, and post-pandemic periods.

While our findings highlight the resilience of HIV care services amidst the COVID-19 pandemic with a strong team of research assistants that ensured the accuracy and completeness of VL, CD4, and ART initiation data, limitations included delays in viral load assessment among individuals enrolled during Period 2 highlight the importance of optimizing healthcare delivery systems to minimize disruptions in routine monitoring and follow-up care during public health crises. Ongoing efforts to strengthen community-based healthcare initiatives and promote self-management strategies among individuals living with HIV can enhance resilience to future pandemics or health emergencies.

## Conclusion

Our study provides valuable insights into the impact of COVID-19 public health measures on HIV viral load suppression among Ugandans living with HIV in serodifferent relationships. Despite facing unprecedented challenges, we saw no meaningful changes in the level of HIV viral suppression in a large sample of PLHIV. The resilience of HIV care services and the commitment of patients and healthcare providers likely played a role in ensuring the continuity of essential treatment and care during this time. Investments in innovative healthcare delivery models and community-based interventions will be essential to strengthen pandemic preparedness and safeguard gains made for HIV care.
